# The Influence of Shape Memory Alloy Volume Fraction on the Impact Behavior of Polymer Composites

**DOI:** 10.3390/polym10111280

**Published:** 2018-11-16

**Authors:** Min Sun, Xiaokun Sun, Zhenqing Wang, Mengzhou Chang, Hao Li

**Affiliations:** College of Aerospace and Civil Engineering, Harbin Engineering University, Harbin 150001, China; sunmin@hrbeu.edu.cn (M.S.); sunxiaokun@hrbeu.edu.cn (X.S.); changmengzhou@hrbeu.edu.cn (M.C.); lihao0202@hrbeu.edu.cn (H.L.)

**Keywords:** polymer composites, shape memory alloy, low-velocity impact

## Abstract

The low-velocity impact behavior of Shape Memory Alloy (SMA) reinforced resin matrix polymers is investigated and the influence of the SMA volume fraction on the impact performance of polymer composites is considered for the first time, which are the highlights in this paper. Firstly, 12 kinds of polymer composite specimens with different SMA volume fractions are fabricated in terms of the SMA layup spacing, SMA diameter, and the interaction between the two. Secondly, a low-velocity impact test is carried out in order to study the impact performances of the above polymer composites. Finally, the damage morphology of the specimen after impact is observed by the visualization method and the low-velocity impact performance of the 12 kinds of polymer composites is analyzed on the basis of the force and energy history curve.

## 1. Introduction

Recently, composite materials are used extensively as a result of their inherently high specific mechanical properties as well as low weight. However, due to the lack of a thorough thickness reinforcement or weak interfaces of the composites, which often produce some visible or invisible damage in structures subjected to external loads, these damages will greatly affect the mechanical performance, and reduce the integrity and stability of composite structures, and then reduce the lifetime of composite materials [1,2,3,4,5,6,7,8,9]. Therefore, many researchers commit to finding the possible ways to improve load carrying capacity and reduce the damage of composites. In 1963, Buehler et al. [10] made it clear that shape memory alloy (SMA) can exhibit a unique mechanical memory, and since then SMAs have been the research focus of scholars, especially as a reinforcement material. Due to the unique phase transformation behavior, SMAs exhibit an excellent super-elastic behavior, large recoverable deformation, good fatigue life, and low power consumption [11,12,13,14]. Therefore, embedding SMA into composites becomes a very important way to improve the damage resistance properties of composite structures.

Over the past years, a considerable amount of research has been devoted to evaluating the mechanical and impact performances of SMA and fiber hybrid reinforced composites. Zhang et al. [15] studied the mechanical properties of composites filled with SMA particles and short fibers. Zhou et al. [16] experimentally evaluated the bending behavior of composite beams embedded with SMA wires. Taheri et al. [17] studied the characterization of a shape memory alloy hybrid composite plate subjected to static loading and discussed the influence of various parameters on the overall behavior of the shape memory alloy hybrid composite (SMAHC) plate. Lei et al. [18] investigated the macroscopic mechanical behavior of SMA hybrid composites with a weak interface under quasi-static loading. Furthermore, Paine et al. [19] studied the response of SMA hybrid composite materials subjected to a low-velocity impact and examined a new concept of composite material toughening. Roh et al. [20] optimized SMA volume fractions of smart composite plates under low-velocity impact. Tsoi et al. [21] investigated the impact damage behavior of SMA composites. Lau et al. [22] studied the damage resistance properties of SMA embedded glass/epoxy composites after a low-velocity impact. Khalili et al. [23] investigated the effect of some important parameters on the low-velocity impact response of the active thin walled hybrid composite plates embedded with SMA. Pappadà et al. [24] showed the influence of the integration of SMA to suppress the propagating damage of composite structures. Sun et al. [25] experimentally optimized the influence of different SMAs positions on the impact behavior of GF/epoxy laminates. Besides, Meo et al. [26] wrote a review on improving the impact properties of SMA hybrid composites in aeronautical applications. Kang et al. [27] also identified the effect of SMA on the damage behavior of laminates subjected to low-velocity impacts at low temperatures of 293, 263, and 233 K.

Although the mechanical and impact performances of SMA and fiber hybrid reinforced composites have been an active research for many years, there are only few studies in the literatures on SMA separately reinforced resin matrix composites, especially that of impact behavior. Raghavan et al. [28] studied the damping, tensile, and impact properties of super-elastic SMA fiber-reinforced polymer composites. It was found that an appreciable improvement can be observed in damping, tensile, and impact properties of the polymer matrix due to reinforcement with SMA fibers. Wang et al. [29,30] investigated the internal stress distribution in a single SMA fiber-matrix composite. Lei et al. [31] experimentally studied and numerically the interface between the SMA fiber and matrix based on pull-out tests and uniaxial tensile tests, and the cohesive zone model approach. Liu et al. [32] studied the interface performance between shape memory alloy fibers and the polymer matrix using a silane coupling agent KH550 and Al_2_O_3_ nanoparticles.

This paper focuses on the low-velocity impact behavior of SMA separately reinforced resin matrix polymer composites based on the above research. More importantly, the influence of the SMA volume fraction on the impact performance of polymer composites is studied for the first time. For these goals, 12 kinds of polymer composites with different SMA volume fractions are fabricated and a low-velocity impact test is conducted.

## 2. Experimental

### 2.1. Materials and Manufacturing

**Figure 1:**
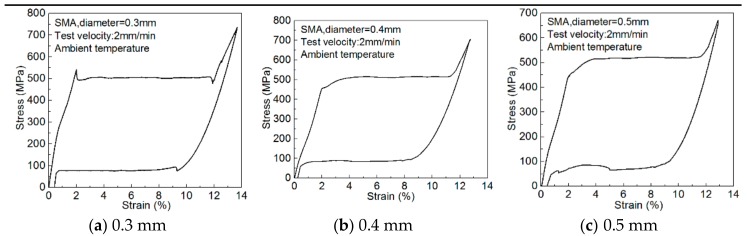
The stress–strain diagram of Ni-Ti shape memory alloy wires.

The polymer matrix is synthesized by an epoxy vinyl ester resin of bisphenol A (Derakane Momentum 411, Dow, Midland, MI, USA), methyl ethyl ketone peroxide (Butanox M-50, Dow) as the hardener and Dimethylaniline (ZF-IV, Dow) as the accelerator in the weight ratio of 100:1.5:0.15. All the chemical products are commercially available and used as received without further purification. The shape memory alloy (SMA) wire is a super-elastic 55.71%, 55.71%, and 56.09% Ni balanced with Ti alloy with 0.3-, 0.4-, and 0.5-mm diameters, respectively, and their basic material parameters (provided by Jiangyin Fasten-PLT Materials Science Co. Ltd., Jiangyin, Jiangsu, China) are shown in Table 1. The stress–strain curves (determined by mechanical experiments) are shown in Figure 1. All the as received Ni-Ti wires are covered with a thin oxide layer. Before use, the Ni-Ti wire surface is polished with sandpaper to remove the thin oxide layer, and then it is wiped with degreased gauze soaked in acetone to remove surface stains.

**Figure 2:**
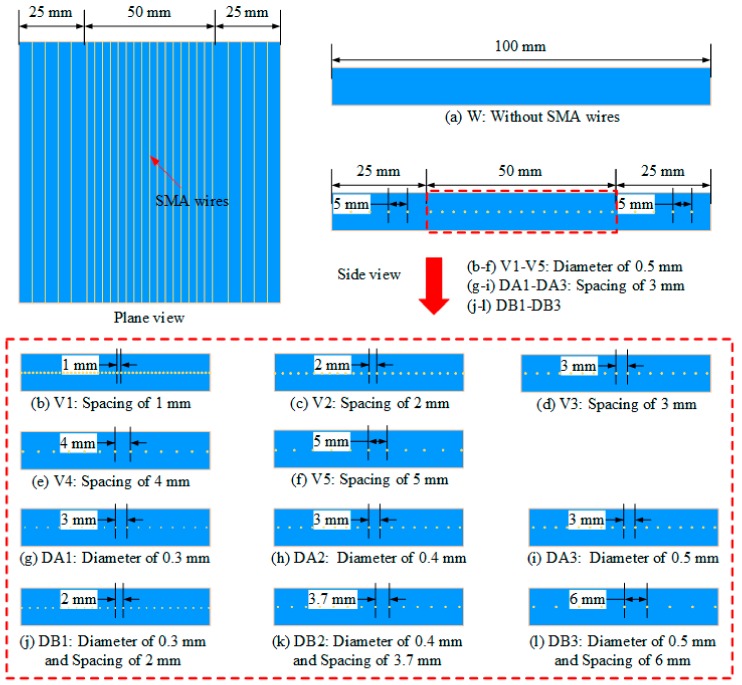
The schematics and codes of composites specimens.

All composites specimens are divided into 12 groups, with the schematics and codes shown in Figure 2 and the SMA volume fractions (VF) in the 12 group composite specimens shown in Table 2. The first group (code: W) is pure epoxy resin matrix composites, without SMA wires. The second to sixth groups (code: V1/V2/V3/V4/V5) are SMA reinforced composites embedded with an SMA diameter of 0.5 mm and SMA layup spacing values of 1, 2, 3, 4, 5 mm, respectively. The seventh to ninth groups (code: DA1/DA2/DA3) are SMA reinforced composites embedded with an SMA layup spacing of 3 mm and SMA diameters of 0.3, 0.4, 0.5 mm, respectively. The tenth to twelfth group (code: DB1/DB2/DB3) are SMA reinforced composites embedded with SMA diameters of 0.3, 0.4, 0.5 mm, and SMA layup spacing values of 2, 3.7, 6 mm, respectively. The layup spacing in all the above SMA reinforced specimens refer to that of an engraved area and the layup spacing in the non-encrypted area is 5 mm. The SMA diameter in the same group in the engraved area and the non-encrypted area is the same.

**Figure 3:**
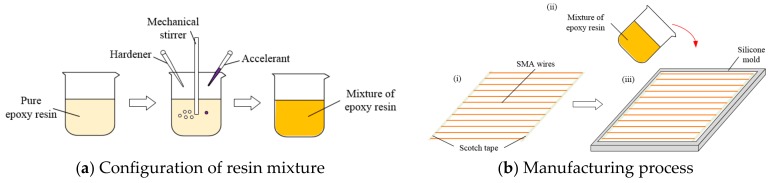
The overall preparation process of the SMA reinforced polymer composites.

The overall preparation process of SMA-reinforced polymer composites is shown in Figure 3, which mainly includes the following 3 steps:

(i) The treated SMA wire is arranged neatly according to the above design scheme (Figure 2), and the two ends of all SMA wires are fixed by using a scotch tape, respectively, and then they are placed in a silicon mold.
(ii) The epoxy resin is mixed, the curing agent and the accelerator in a ratio of 100:1.5:0.15 is added, then they are uniformly stirred, and placed in a vacuum drying oven for about 4 min to extract the air. Note: The vacuum time should not be too long, otherwise the mixture will easily become gelled, resulting in the failure of the preparation.
(iii) The air-free mixtures are poured into the mold with SMA wires, and the whole system is cured at room temperature for 12 h.

Besides, we also give the marketing cost of the various types of polymer composites, as seen in Table 3.

### 2.2. Low-Velocity Impact Test

**Figure 4:**
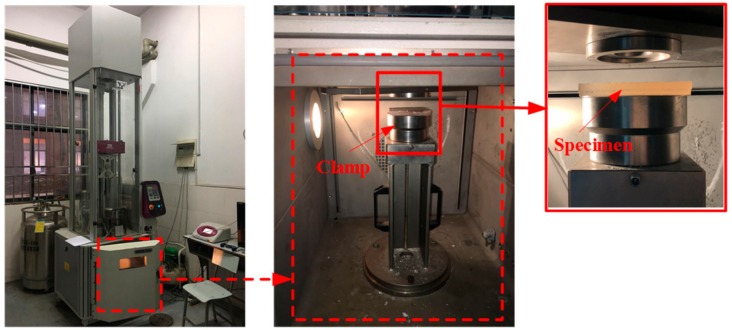
The Instron CEAST 9350HV drop weight impact testing machine.

Low-velocity impact tests are performed by using an Instron CEAST 9350HV drop weight impact testing machine (Tongji, Shanghai, China) (Figure 4) according to ASTM D5420-2010. The mass and diameter of the hemispherical impactor are 3.777 kg and 16 mm, respectively. The diameter of the circular ring clamps is 76 mm, and the geometries of all specimens are 100 mm × 100 mm × 10 mm. All the above received composites need to be polished using a metallographic sample grinding machine to make the specimen smooth and flat.

The impact behavior of composites subjected to the impact energy of 5 and 14 J is tested. The velocity-time and displacement-time curves of the impactor were calculated in terms of ASTM D5420-2010. The force–time curve of the impactor can be captured by a high accuracy sensor. The absorbed energy E(t) of the composites at a certain time *t* during the impact process can be calculated by Equation (1)

(1) $$E(t)=\frac{1}{2}mv_{0}^{2}-\frac{1}{2}mv^{2}(t)+mg\delta (t)$$

where *m* is the mass of impactor, *v*_0_ is the initial velocity of the impactor, the gravitational acceleration *g* is 9.81 m/s^2^, and *v*(*t*) and *δ*(*t*) are the velocity and displacement of the impactor at time *t*, respectively.

## 3. Results and Discussion

In order to better observe the damage morphology of the 12 group composites under low-velocity impact, the damage graph of the specimen after impact (Section 3.1) and the force and energy curve of the specimen after impact (Section 3.2) are analyzed and discussed.

### 3.1. Failure Modes of Composites after the Low-Velocity Impact Test

#### 3.1.1. Composites with Different SMA Layup Spacing Value

**Figure 5:**
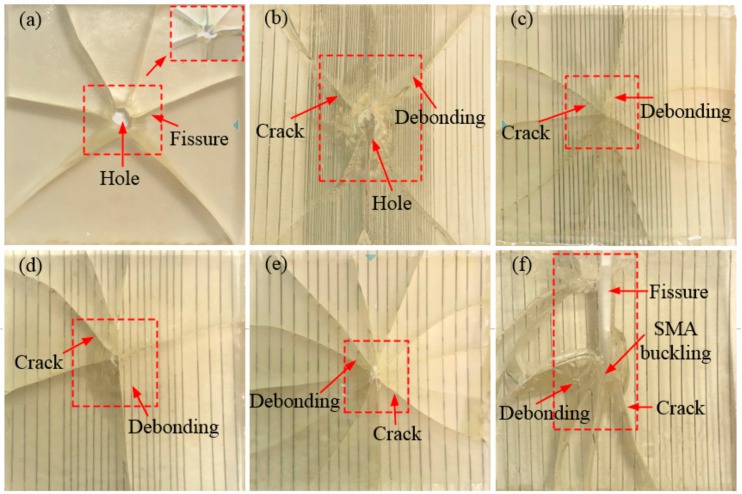
The failure modes diagrams of specimens with different SMA layup spacing values under 5 J of energy: (**a**) indicates the pure resin-matrix test specimen; (**b**–**f**) indicate the SMA reinforced resin-matrix test specimens with SMA layup spacing of 1, 2, 3, 4, 5 mm, respectively.

**Figure 6:**
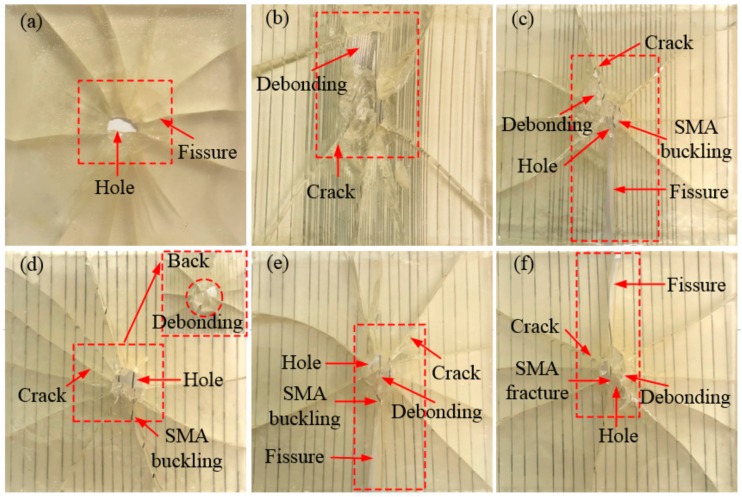
The failure modes diagrams of spacing specimens with different SMA layup spacing under 14 J of energy: (**a**) indicates the pure resin-matrix test specimen; (**b**–**f**) indicate the SMA reinforced resin-matrix test specimens with SMA layup spacing of 1, 2, 3, 4, 5 mm, respectively.

Figure 5 and Figure 6 show the failure mode diagrams of 6 groups composites with different SMA layup spacing under the two impact energies of 5 and 14 J, respectively. In the three figures, (a) is a pure resin-matrix test specimen (code: W); (b)–(f) are SMA reinforced resin-matrix test specimens with SMA layup spacing of 1, 2, 3, 4, 5 mm (code: V1, V2, V3, V4, V5).

It can be seen from Figure 5 that the impact position of the pure resin-matrix test specimen is penetrated, cracks appear in the test specimen, and both sides of the test specimen are completely split at the crack under 5 J of impact energy. However, most of SMA reinforced resin-matrix test specimens only show cracks and both sides of the test specimens at the crack are still a whole. That is, the specimen embedded in the SMA has an improved integrity and reduced damage under 5 J of impact energy, which indicates that the embedding SMA can greatly improve the impact resistance performance of the test specimen. For the test specimen with an SMA layup spacing of 1 mm (Figure 5b), there is a penetration hole at the impact position of the test specimen, the test specimen has a small number of cracks, and the crack basically expands from the impact position to the four sides of the test specimen. Besides, the debonding between the alloy and resin occurs around the crack. The specimen with an SMA layup spacing of 2 mm (Figure 5c) showed no penetrating hole at the impact position of the specimen, only a small amount of crack appeared in the specimen, and the debonding between the alloy and resin also occurred around the crack. For the test specimen with an SMA laying pitch of 3 mm (Figure 5d), a small piece resin fell off at the impact position of the test specimen, a small amount of cracks appeared in the test specimen, and there was also debonding between the alloy and resin that had occurred around the crack. For the test specimen with an SMA laying pitch of 4 mm (Figure 5e), the specimen shows more cracks and there was obviously a debonding phenomon between the alloy and resin. For the test specimen with an SMA laying pitch of 5 mm (Figure 5f), the damage in the specimens was serious, there were no more cracks and alloy buckling phenomena, and there was also a long crack parallel to the direction of the alloy on both sides of the test specimen. This shows that the layup spacing of the SMA had a great influence on the overall damage of the test specimen. The impact resistance of the test specimen with the SMA layup spacing of 2, 3, and 4 mm is better, and that of the test with the SMA layup spacing of 1 and 5 mm is poor.

As can be seen from Figure 6, when the impact energy is increased to 14 J, all the test specimens were substantially completely penetrated compared to the 5 and 10 J of impact energy. For the pure resin matrix test specimen (Figure 6a), the test specimen showed more cracks and the test specimen is completely separated at both sides of the crack and the whole test specimen is almost not the bearing capacity. For the test specimen with an SMA layup spacing of 1 mm (Figure 6b), there is a large penetration hole at the impact position of the test specimen, the debonding phenomenon between the alloy and the resin is serious in the encryption zone, and the resin is almost completely dropped. For the test specimen with an SMA layup spacing of 2 mm (Figure 6c), a large penetration hole appears at the impact position of the test specimen, and, at the same time, the alloy buckling phenomenon appears and more cracks appear in the test specimen. Besides, a long crack extends from the impact position to both sides of the specimen, the direction of the crack is parallel to the direction of the alloy embedded, and the test specimen is completely separated at both sides of the crack. For the test specimen with an SMA layup spacing of 3 mm (Figure 6d), a large penetrating hole appears at the impact position of the test specimen, and at the same time, the alloy buckling phenomenon appears, and more cracks appear in the whole test specimen. In addition, the back damage diagram of the specimen under an impact energy of 14 J is also shown in the upper right corner of Figure 6d. It can be seen that the debonding phenomenon between the alloy and the resin at the impact position is serious. For the test specimen with an SMA layup spacing of 4 mm (Figure 6e), there is a large penetration hole at the impact position, accompanied by an alloy buckling phenomenon and more cracks. In addition, a long crack extends from the impact position to one side of the specimen and the test specimen is completely separated at both sides of the crack. For the test specimen with an SMA layup spacing of 5 mm (Figure 6f), a large penetration hole appears at the impact position and the alloy is broken. In addition, a long crack extends from the impact position to one side of the specimen and the test specimen is completely separated at both sides of the crack. Compared with that under the impact energy of 5 J, the damage of all the specimens is more serious under the impact energy of 14 J.

However, the overall damage trend of different types of test specimens is basically the same under the three impact energies of 5 and 14 J. The impact resistance of the test specimens with SMA layup spacing of 2, 3, and 4 mm is better, the SMA layup spacing of 1 mm is good, and the SMA layup spacing of 5 mm is poor.

#### 3.1.2. Composites with Different SMA Diameters

**Figure 7:**
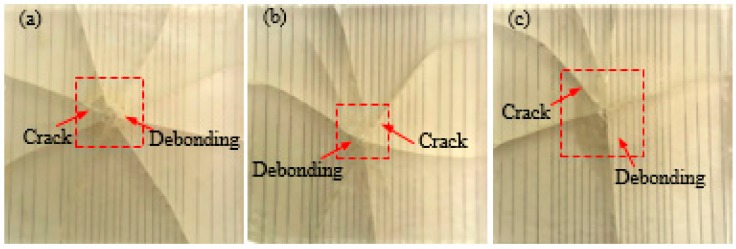
The failure modes diagrams of specimens with different SMA diameters under 5 J of energy: (**a**–**c**) indicate the SMA reinforced resin-matrix test specimens with SMA diameters of 0.3, 0.4, 0.5 mm, respectively.

**Figure 8:**
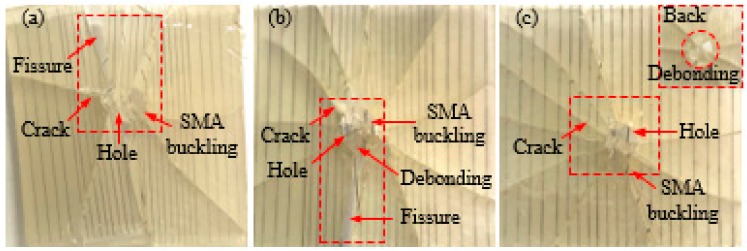
The failure modes diagrams of specimens with different SMA diameters under 14 J of energy: (**a**–**c**) indicate the SMA reinforced resin-matrix test specimens with SMA diameters of 0.3, 0.4, 0.5 mm, respectively.

Figure 7 and Figure 8 show the failure mode diagrams of 3 group composites with different SMA diameters under spacing two impact energies of 5 and 14 J, respectively.

It can be seen from Figure 7 that a small number of cracks appear in the three types of specimens under 5 J of impact energy and the difference between the number of cracks in the three types of specimens is not very significant. This shows that the overall damage state of the test specimen is not affected by the diameter of the SMA when 5 J of impact energy is applied.

As can be seen from Figure 8, when the impact energy is increased to 14 J, the damage of all the test specimens is more serious than the impact energy of 5 J. For the test specimen embedded SMA diameter of 0.3 mm (Figure 8a), there is a large penetration hole at the impact position of the test specimen, accompanied by the alloy buckling phenomenon and more cracks. In addition, a long crack extends from the impact position to one side of the spacing specimen and the test specimen is completely separated at both sides of the crack. For the test specimen embedded SMA diameter of 0.4 mm embedded in the SMA (Figure 8b), a large penetration hole appears at the impact position of the test specimen, accompanied by more cracks. Besides, a long crack also extends from the impact position to one side of the spacing specimen and the test specimen is completely separated at both sides of the crack. For the test specimen embedded SMA diameter of 0.5 mm (Figure 8c), there is a larger penetration hole at the impact position of the test specimen, accompanied by the alloy buckling phenomenon and more cracks. Furthermore, the back damage diagram of the specimen under an impact energy of 14 J is also shown in the upper right corner of Figure 8c. It can be seen that the debonding phenomenon between the alloy and the resin at the impact position is serious.

In summary, from the overall damage of the test specimen, the diameter of the SMA has a certain influence on the overall damage state of the test specimen. The damage of the test specimen embedded with an SMA diameter of 0.5 mm is less than that of embedded SMA diameters of 0.3 and 0.4 mm.

#### 3.1.3. Composites with Different SMA Diameters and Layup Spacing Values

**Figure 9:**
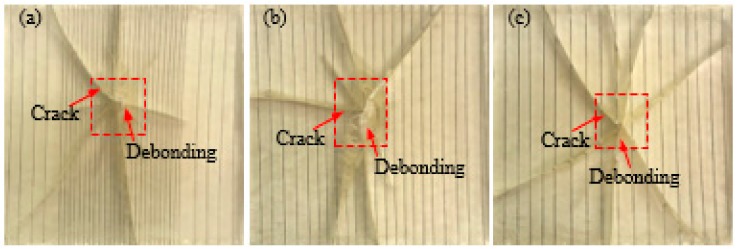
The failure modes diagrams of specimens with different SMA layup spacing values and diameters under 5 J of energy: (**a**–**c**) indicate the SMA reinforced resin-matrix test specimens with SMA diameters of 0.3, 0.4, 0.5 mm, respectively, and SMA layup spacing of 2, 3.7, 6 mm, respectively.

**Figure 10:**
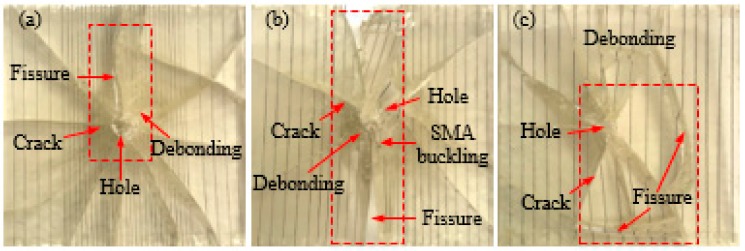
The failure modes diagrams of specimens with different SMA layup spacing values and diameters under 14 J of energy: (**a**–**c**) indicate the SMA reinforced resin-matrix test specimens with SMA diameters of 0.3, 0.4, 0.5 mm, respectively, and SMA layup spacing of 2, 3.7, 6 mm, respectively.

Figure 9 and Figure 10 show the failure mode diagrams of 3 group composites with different SMA diameters and SMA layup spacing under spacing two impact energies of 5 and 14 J, respectively.

It can be seen from Figure 9 that a small number of cracks appear in the three groups specimens, and the debonding between the alloy and the resin around the crack appear under 5 J of impact energy. The specimen (DB1) with an SMA layup spacing of 2 mm and an alloy diameter of 0.3 mm has fewer cracks (Figure 9a), and more cracks appear in the specimen (DB3) with an SMA layup spacing of 6 mm and an alloy diameter of 0.5 mm (Figure 9c). This shows that the effect of the SMA layup spacing on the overall damage state of the test specimen is greater than that of the SMA diameter when 5 J of impact energy is applied.

As can be seen from Figure 10, when the impact energy is increased to 14 J, the damage of all the test specimens is more serious than the impact energy of 5 J. For the test specimen with an SMA layup spacing of 2 mm and an alloy diameter of 0.3 mm (Figure 10a), a large penetration hole appears at the impact position of the test specimen, a long crack extends from the impact position to one side of spacing specimen, and the test specimen is completely separated at both sides of the crack. For the test specimen with an SMA layup spacing of 3.7 mm and an alloy diameter of 0.4 mm (Figure 10b), there is also a large penetration hole at the impact position of the specimen, accompanied by alloy buckling. Besides, a long crack extends from the impact position to one side of the specimen and the test specimen is completely separated at both sides of the crack. For the test specimen with an SMA layup spacing of 6 mm and an alloy diameter of 0.5 mm (Figure 10c), the whole test specimen produces more damage and two long cracked gaps appear, which is in parallel and in a perpendicular direction to the alloy.

In summary, the SMA layup spacing has a greater influence on the overall damage state of the test specimen than that of the SMA diameter when 14 J of impact energy is applied.

### 3.2. Influence of SMA Volume Fractions on Spacing Impact Behavior of spacing Composite

#### 3.2.1. Different SMA Volume Fractions (Different SMA Layup Spacing)

**Figure 11:**
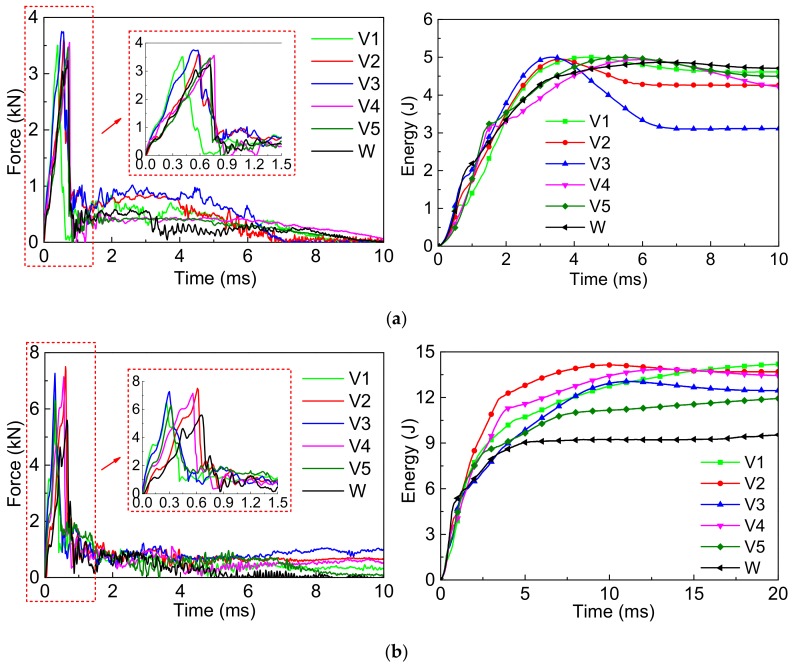
The force-time curves and energy-time curves of the composites with different SMA spacing under two impact energies: (**a**) Initial impact energy of 5 J; (**b**) Initial impact energy of 14 J.

Figure 11 shows the force–time (*F-t*) history curves and energy–time (*E-t*) history curves of SMA reinforced resin matrix composites embedded with an SMA diameter of 0.5 mm and different SMA layup spacing values of 1, 2, 3, 4 and 5 mm respectively under two impact energies.

From *F-t* curves of Figure 11a, the overall variation tendency of the contact force curves of 6 groups specimens are relatively consistent under 5 J of impact energy, and they all increase first and then decrease. The difference is that there is a drop in the loading stage of spacing contact force curve of the spacing specimen without SMA, however, the loading stage curve of the contact force of specimens with SMA is relatively smooth. In addition, it can be also concluded that the peak force of specimen V3 is the highest with a value of 3.75 kN, and that of specimen W with a value of 3.23 kN is the lowest among spacing 6 groups specimens. From *E-t* curves of Figure 11a, the overall variation tendency of the absorbed energy curves of all specimens are relatively consistent under 5 J of impact energy. During the impact process, the impact energy of the impactor is firstly converted into the absorbed energy of the spacing specimen and then a part of spacing absorbed energy is released, which is the recoverable absorbed energy. Finally, the absorbed energy reaches a stable value, which is the ultimate absorbed energy. The absorbed energy is mainly from the impact damage of spacing specimen and the absorbed energy of SMA. Furthermore, the ultimate absorbed energy of the specimen V3 is the smallest with a value of 3.11 J, which represents that the recoverable absorbed energy is the highest among the 5 groups specimens with SMA. It also indicates that the super-elastic property of SMA of specimen V3 plays the best role among spacing above specimens. The ultimate absorbed energy of specimen W with a value of 4.71 J is the largest and the impact energy is almost completely absorbed by spacing damage of spacing specimen.

From the *F-t* curves of Figure 11b, a drop occurs in the loading stage curves of the contact force of all specimens under 14 J of impact energy and the peak forces of specimens V2/V3/V4 with values of 7.50, 7.26, and 7.14 kN, respectively, are relatively the highest. From the *E-t* curves of Figure 11b, the absorbed energy curves of all specimens almost directly reach a stable platform and it represents that the recoverable absorbed energy of all specimens is smaller. It also indicates that most of the impact energy is absorbed by the damage of the specimen and less is absorbed by the SMA under 14 J of impact energy. Similarly, it also can be found that the recoverable absorbed energy is the highest in specimen V3 under 14 J of impact energy.

**Figure 12:**
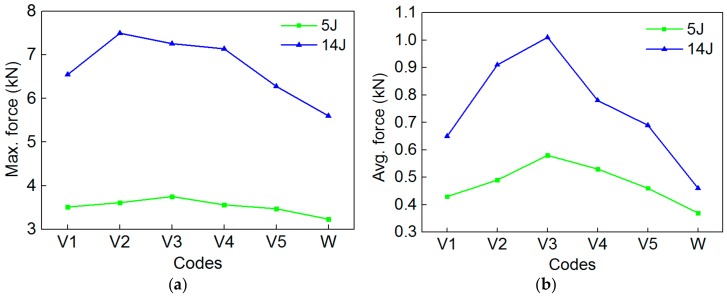
The maximum contact force and average contact force value of specimens of V1/V2/V3/V4/V5/W under the two impact energies: (**a**) Max. force; (**b**) Avg. force.

From the *F-t* curves of Figure 11, we see that despite the loading and unloading phases of the specimen having a short duration time, the contact force between the impact head and specimen always exists during the impact process. Therefore, to more accurately optimize the SMA layup spacing, the comparison of the peak contact force and the average contact force of the specimen are shown in Figure 12. The trends of the maximum contact force (Figure 12a) and the average contact force (Figure 12b) of the 6 groups specimens are consistent under the two impact energies. Comparing Figure 12a,b, all the trends of the maximum contact force and average contact force of the 6 groups specimens are also consistent under the same impact energy. Furthermore, the trend of the average contact force is more intuitive than that of the maximum contact force. The maximum contact force and the average contact force of specimens V2/V3/V4 are relatively larger. This indicates that embedding different SMA layup spacing gas a great influence on the load carrying capacity of the specimen. This may be due to the adhesion between the alloy and resin of the specimen that presents some difference when embedding different SMA layup spacing values. A reasonable SMA layup spacing is beneficial for playing the super-elastic properties of SMA, thus, improving the impact resistance of the composites.

In order to more clearly illustrate the specific value of the impact parameters of the 6 groups composite specimens, the maximum contact force, the average contact force, and the ultimate absorbed energy of the composites with different SMA layup spacing values are shown in Table 4.

#### 3.2.2. Different SMA Volume Fractions (Different SMA Diameters)

**Figure 13:**
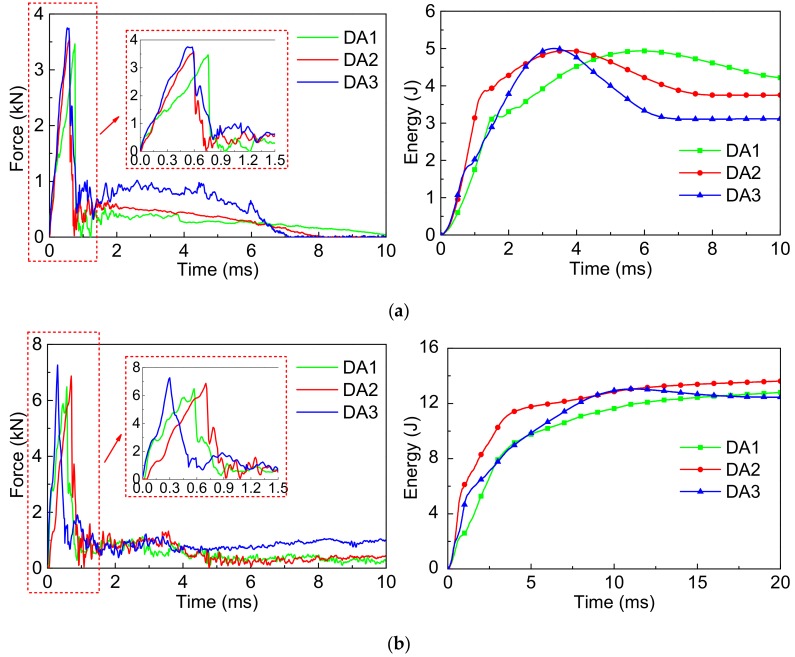
The force-time curves and energy-time curves of composites with different SMA diameters under the two impact energies: (**a**) Initial impact energy of 5 J; (**b**) Initial impact energy of 14 J.

Figure 13 shows the *F-t* and *E-t* curves of SMA reinforced resin matrix composites with an SMA layup spacing of 3 mm and different SMA diameters of 0.3, 0.4, and 0.5 mm respectively under the two impact energies.

From the *F-t* curves of Figure 13a, the overall variation tendency of the contact force curves of the 3 group specimens are relatively consistent under 5 J of impact energy. Specimen DA3 has the highest peak force with a value of 3.75 kN, and that of specimen DA1 with a value of 3.46 kN is the smallest. From the *E-t* curves of Figure 13a, the overall variation tendency of the absorbed energy curves of the 3 group specimens are relatively consistent under 5 J of impact energy. The ultimate absorbed energy of specimen DA3 with a value of 3.11 J is the smallest, which represents the recoverable absorbed energy which is the most among the 3 group specimens. The ultimate absorbed energy of specimen DA1 with a value of 4.22 J is the largest, which has the least recoverable absorbed energy.

From the *F-t* curves of Figure 13b, the peak force of specimen DA3 with a value of 7.26 kN is the highest and specimen DA1 with a value of 6.48 kN is the lowest under 14 J of impact energy. From the *E-t* curves of Figure 13b, the absorbed energy curves of the 3 group specimens almost directly reach a stable platform under 14 J of impact energy, and the recoverable absorbed energy of the 3 group specimens is significantly reduced. Similarly, the recoverable absorbed energy of specimen DA3 is the most and that of specimen DA1 is the least.

**Figure 14:**
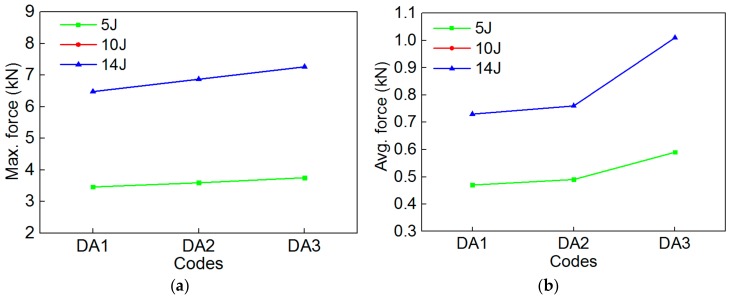
The maximum contact force and average contact force value of the specimen of DA1/DA2/DA3 under the two impact energies: (**a**) Max. force; (**b**) Avg. force.

Similarly, to more accurately study the effect of the SMA diameters on the impact behavior of the composites, the comparison of the maximum contact force and average contact force of the specimen are shown in Figure 14. The trends of the maximum contact force (Figure 14a) and average contact force (Figure 14b) of the 3 group specimens are consistent under the two impact energies. Comparing Figure 14a,b, the whole trends of the maximum contact force and average contact force of the 3 group specimens are also consistent under the same impact energy, furthermore, the trend of the average contact force is more intuitive than that of the maximum contact force. Specimen DA3 has the highest maximum contact force and the average contact force among the 3 group specimens. This indicates that embedding different SMA diameters has a great influence on the load carrying capacity of the specimen. This is because different SMA diameters lead to a difference of the contact area between the impact head and SMA during the impact process, thus, resulting in the difference of the load carrying capacity of the 3 group specimens. A reasonable SMA diameter is beneficial to playing the super-elastic properties of SMA, thus, improving the impact resistance of the composites.

In order to more clearly illustrate the specific value of the impact parameters of the 3 group composite specimens, the maximum contact force, the average contact force, and the ultimate absorbed energy of composites with different SMA diameters are shown in Table 5.

#### 3.2.3. Different SMA Volume Fractions (Different SMA Diameters and Layup Spacing)

**Figure 15:**
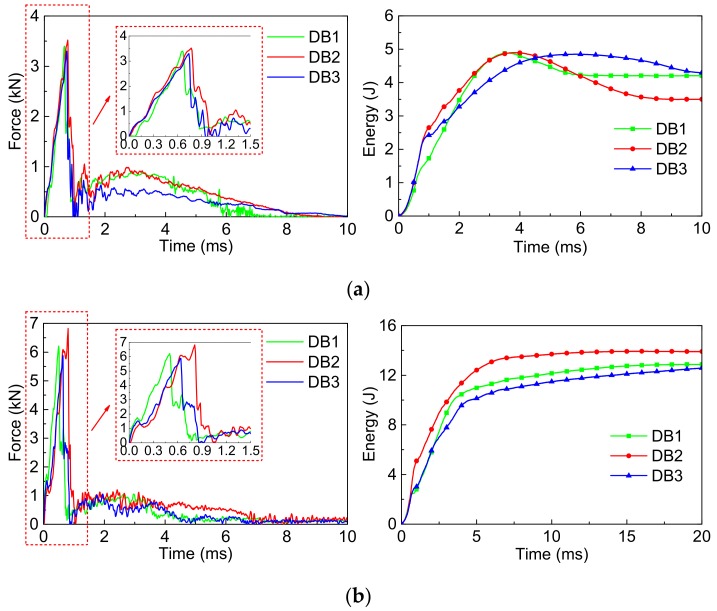
The force-time curves and energy-time curves of composites with different SMA diameters and layup spacing under two impact energies: (**a**) Initial impact energy of 5 J; (**b**) Initial impact energy of 14 J.

Figure 15 shows the *F-t* and *E-t* curves of the SMA reinforced resin matrix composites with different SMA diameters of 0.3, 0.4, and 0.5 mm and SMA layup spacing values of 2, 3.7, and 6 mm, respectively, under the two impact energies.

From the *F-t* curves of Figure 15a, the overall variation tendency of the contact force curves of the 3 group specimens are relatively consistent under 5 J of impact energy. The specimen DB2 with a value of 3.52 kN has the highest peak force, and that of specimen DB3 with a value of 3.30 kN is the lowest. From the *E-t* curves of Figure 15a, the overall variation tendency of the absorbed energy curves of the 3 group specimens are relatively consistent under 5 J of impact energy. The ultimate absorbed energy of specimen DB2 with a value of 3.50 J is the smallest, which represents the recoverable absorbed energy is the most among 3 group specimens. The ultimate absorbed energy of specimen DB3 with a value of 4.29 J is the largest, which has the least recoverable absorbed energy.

From the *F-t* curves of Figure 15b, the peak force of specimen DB2 with a value of 6.82 kN is the highest, and specimen DB3 with a value of 5.88 kN is the lowest under 14 J of impact energy. From the *E-t* curves of Figure 15b, the absorbed energy curves of the 3 group specimens almost directly reach a stable platform under 14 J of impact energy, and the recoverable absorbed energy of 3 group specimens is significantly reduced. Similarly, the recoverable absorbed energy of specimen DB2 is the most, and that of specimen DB3 is the least.

**Figure 16:**
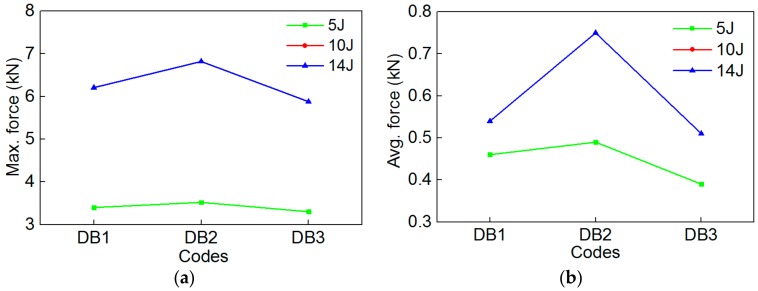
The maximum contact force and average contact force value of DB1/DB2/DB3 specimens under the two impact energies: (**a**) Max. force; (**b**) Avg. force.

Similarly, to more accurately study the impact behavior of composites under different SMA diameters and layup spacing values, the comparison of the maximum contact force and average contact force value of specimens are shown in Figure 16. The trends of maximum contact force (Figure 16a) and average contact force (Figure 16b) of the 3 group specimens are consistent under the two impact energies. Comparing Figure 16a,b, the whole trends of maximum contact force and average contact force of the 3 group specimens are also consistent under the same impact energy. Furthermore, the trend of the average contact force is more intuitive than that of the maximum contact force. The specimen DB2 has the highest maximum contact force and average contact force among the 3 group specimens. It is concluded that the impact behavior of the composites is affected by the diameter and layup spacing of the SMA. As for the above two factors, which has a larger effect on the impact behavior of the specimen, a lot of experimental investigations are needed.

In order to more clearly illustrate the specific value of impact parameters of the 3 groups of composite specimens, the maximum contact force, the average contact force, and the ultimate absorbed energy of the composites embedded with different SMA diameters and layup spacing values are shown in Table 6.

## 4. Conclusions

This paper mainly studies the low-velocity impact performance of SMA-reinforced polymer composites embedded in different layup spacing (1, 2, 3, 4, 5 mm) and diameter (0.3, 0.4, 0.5 mm) values. The influence of alloy spacing and diameter on the damage morphology and impact parameters of the specimens was compared with the impact properties of pure polymer composites. In addition, this chapter extends the difference in the spacing and diameter of the alloy to the influence of the difference in volume fraction on the impact characteristics of the specimen, and further analyzes the impact of the different layup spacing and diameter on the impact characteristics of the specimen based on the volume fraction.

The following conclusions can be drawn:

(1) Embedding the SMA wire into the pure polymer composites can effectively improve the bearing capacity and energy absorption characteristics of the entire test specimen and reduce the damage of the test specimen.
(2) The SMA layup spacing has a great influence on the impact performance of the specimen. The specimen (V3) with a layup spacing of 3 mm has the best impact performance, the largest load carrying capacity and the smallest damage among six groups polymer composites. Here, the impact performance is the best when the volume fraction of the SMA wire in the test specimen is 0.49% in the 3 groups of polymer composites.
(3) SMA diameter also has an effect on the impact properties of the specimen, which is the best for the specimen (DA3) with a diameter of 0.5 mm among the 3 group composite specimens. Here, the impact performance is the best when the volume fraction of the SMA wire in the test specimen is 0.49% in the 3 groups of polymer composites.
(4) When the two factors of the SMA layup spacing and diameter are changed at the same time, the overall damage state of the test specimen, as well as the bearing capacity and energy absorption characteristics are also affected. Among the three test specimens, the impact resistance of the test specimen (DB2) with an SMA layup spacing of 3.7 mm and a diameter of 0.4 mm is the best, and the SMA layup spacing had a greater influence on the impact characteristics of the test specimen than the SMA diameter. Here, the impact performance is best when the volume fraction of the SMA wire in the test specimen is 0.28% in the 3 groups of polymer composites.

## Figures and Tables

**Table 1:** The basic material parameters of Ni-Ti shape memory alloy wires.

Diameter (mm)	0.301–0.303	0.394–0.398	0.501–0.502
$A_{f}$for Delivery (℃)	9.5	14.2	10
Tensile Strength (MPa)	1566.84	1536.27	1535.88
Upper Plateau Stress (MPa)	541.84	564.12	542.26
Lower Plateau Stress (MPa)	116.01	142.48	110.02
Elongation (%)	12.99	12.62	12.75
Residual Elongation (%)	0.05	0.01	0.15

**Table 2:** The shape memory alloy (SMA) volume fractions (VF) of the 12 groups composite specimens.

Codes	VF (%)	Codes	VF (%)	Codes	VF (%)
W	0	V1	1.14	V2	0.65
V3	0.49	V4	0.41	V5	0.37
DA1	0.18	DA2	0.31	DA3	0.49
DB1	0.23	DB2	0.28	DB3	0.33

**Table 3:** The marketing cost of various types of polymer composites.

Codes	Resin Unit Price (¥/L)	Resin Dosage (L)	SMA Diameter (mm)	SMA Unit Price (¥/m)	SMA Dosage (m)	Total Price (¥)	Cost Increase Rate (%)
W	50	0.125	0	0	0	6.25	0
V1	50	0.125	0.3	1	5.0	11.25	80
V2	50	0.125	0.3	1	2.5	8.75	40
V3	50	0.125	0.3	1	1.7	7.95	27.2
V4	50	0.125	0.3	1	1.3	7.55	20.8
V5	50	0.125	0.3	1	1.1	7.35	17.6
DA1	50	0.125	0.3	1	1.7	7.95	27.2
DA2	50	0.125	0.4	1.5	1.7	8.8	40.8
DA3	50	0.125	0.5	2	1.7	9.65	54.4
DB1	50	0.125	0.3	1	2.5	8.75	40
DB2	50	0.125	0.4	1.5	1.4	8.35	33.6
DB3	50	0.125	0.5	2	0.9	8.05	28.8

**Table 4:** The impact parameters of specimens of V1/V2/V3/V4/V5/W under the two impact energies.

Parameters	Initial Energy	V1	V2	V3	V4	V5	W
Max. contact force (kN)	5 J	3.51	3.61	3.75	3.56	3.47	3.23
	14 J	6.55	7.50	7.26	7.14	6.28	5.60
Avg. contact force (kN)	5 J	0.43	0.49	0.58	0.53	0.46	0.37
	14 J	0.65	0.91	1.01	0.78	0.69	0.46
Ultimate absorbed Energy (J)	5 J	4.6	4.26	3.11	4.22	4.50	4.71
	14 J	12.74	14.13	12.93	13.43	11.16	9.23

**Table 5:** The impact parameters of the specimens of DA1/DA2/DA3 under the two impact energies.

Parameters	Initial Energy	DA1	DA2	DA3
Max. contact force (kN)	5 J	3.46	3.59	3.75
	14 J	6.48	6.87	7.26
Avg. contact force (kN)	5 J	0.47	0.49	0.59
	14 J	0.73	0.76	1.01
Ultimate absorbed Energy (J)	5 J	4.22	3.75	3.11
	14 J	11.63	12.85	12.93

**Table 6:** The impact parameters of the DB1/DB2/DB3 specimens under the two impact energies.

Parameters	Initial Energy	DB1	DB2	DB3
Max. contact force (kN)	5 J	3.4	3.52	3.3
	14 J	6.21	6.82	5.88
Avg. contact force (kN)	5 J	0.46	0.49	0.39
	14 J	0.54	0.75	0.51
Ultimate absorbed Energy (J)	5 J	4.21	3.50	4.29
	14 J	12.88	13.90	12.58
